# The Effect of Dog-Assisted Intervention on Student Well-Being, Mood, and Anxiety

**DOI:** 10.3390/ijerph14050483

**Published:** 2017-05-05

**Authors:** Dasha Grajfoner, Emma Harte, Lauren M. Potter, Nicola McGuigan

**Affiliations:** Department of Psychology, Heriot Watt University, Edinburgh EH14 4AS, UK; emma.harte93@googlemail.com (E.H.); L.M.Potter@hw.ac.uk (L.M.P.); N.McGuigan@hw.ac.uk (N.M.)

**Keywords:** dog-assisted intervention, wellbeing, mood, anxiety, higher education

## Abstract

This novel, exploratory study investigated the effect of a short, 20 min, dog-assisted intervention on student well-being, mood, and anxiety. One hundred and thirty-two university students were allocated to either an experimental condition or one of two control conditions. Each participant completed the Warwick–Edinburgh Mental Well-Being Scale (WEMBS), the State Trait Anxiety Scale (STAI), and the UWIST Mood Adjective Checklist (UMACL) both before, and after, the intervention. The participants in the experimental condition interacted with both the dogs and their handlers, whereas the control groups interacted with either the dog only, or the handler only. The analyses revealed a significant difference across conditions for each measure, with those conditions in which a dog was present leading to significant improvements in mood and well-being, as well as a significant reduction in anxiety. Interestingly, the presence of a handler alongside the dog appeared to have a negative, and specific, effect on participant mood, with greater positive shifts in mood being witnessed when participants interacted with the dog alone, than when interacting with both the dog and the handler. These findings show that even a short 20 min session with a therapy dog can be an effective alternative intervention to improve student well-being, anxiety, and mood.

## 1. Introduction

Animals frequently play a visible role in human society, both in the context of companionship and work [1]. A variety of evidence, both anecdotal [2,3] and empirical, has demonstrated that these human-animal interactions can have a positive impact on human health and well-being, through animal-assisted interventions (AAIs), animal-assisted coaching (AAC) [4], animal-assisted activities (AAA) [5], and more recently animal visitation programs (AVPs) [6]. The vast majority of previous research on AAI and AAA focuses on clinical populations [7]; however, there is a growing body of evidence that suggests that animals can have a positive influence on well-being, and quality of life, in non-clinical populations [8,9,10,11,12].

One non-clinical group that has recently received a great deal of attention in the AAI literature is students, where the impact of animals, especially dogs, is reflected in increasingly popular animal-assisted intervention, and visitation programs, within educational settings, particularly higher education [6]. Student mental health, resilience, and well-being are key concerns for universities as they impact student performance generally, as well as students’ ability to deal with the pressure of being away from home, the ability to integrate into a new environment, and to perform in exam situations, all of which are factors that can cause considerable stress and compromise mental health [13]. Indeed, research indicates that students show significantly lower mental health functioning, and higher levels of stress, in comparison to their non-student peers [14], suggesting that the implementation of effective intervention strategies within higher education are of extreme importance.

The benefits of university led dog-assisted intervention, and visitation, programs are potentially manifold. One such benefit is in encouraging students to perceive counseling services as more accessible, allowing support to be sought early [15,16]. A number of universities have begun to offer animal visitation programs, where students can attend sessions of up to 20 min in duration with therapy dogs [6,17]. The results of such studies have demonstrated positive effects, including a reduction in anxiety and negative mood [6], as well as an increased feeling of love and support [16]. Furthermore, involvement of therapy dogs in weekly AAA sessions increased student perception of their own well-being [18], and decreased psychological stress [19]. The benefit of interacting with animals has also been examined within the context of transition to university, a life event that is reported as highly stressful for many students. Research shows that AAI decreased homesickness and increased well-being [20], with students reporting that therapy dogs represent a source of comfort, acceptance, and de-stress [21]. Importantly, the students reported that animal-assisted therapy programs could replace other forms of therapy, and even help to increase social relationships [22], highlighting the important role that such interventions could play in maintaining well-being in this population.

One aspect of these dog-assisted programs that has yet to be subject to scrutiny is what specific element(s) of the interaction led to the benefit(s) witnessed. Within standard university-led dog-assisted programs (for example, Therapet by Canine Scotland), the interaction is three-way, i.e., between the dog, the handler, and the student. It is therefore unclear whether the students benefited from the interaction with the dog, the interaction with the handler, or the combination of the two. This question is extremely important in maximizing the students’ therapy experience and the benefits of such interactions. Indeed, in previous attempts to dissect these elements, students were found to report that therapy dogs, and to a lesser extent their handlers, offered love and support, which was interpreted by the students as reciprocal positive feelings [16]. In contrast, other research has shown that people under stress benefit equally from the presence of a friendly dog, or a friendly human [23]. The dissection of these different elements of human–animal interaction is therefore a question ripe for further research.

It is with this background that the current study aimed to tease apart the relative influence of dogs, and their handlers, during a short interaction with university students. More specifically, we exposed groups of students to a 20 min interaction with either: (1) a dog and their handler, (2) a dog only, or (3) a handler only. In order to determine which type of interaction, if any, was most beneficial, we recorded the students’ mood, anxiety levels, and well-being both before, and after, their respective interaction. We predicted that the students would benefit most in the two conditions where a dog was present, with the handler-only condition being the least beneficial. However, it was less clear whether interacting with both the handler and the dog would result in greater improvements within the student group than interacting with the dog only. In spite of this, we predicted that, if the presence of a dog was key, then both conditions where a dog was present would be equally beneficial. We did however concede that it was possible that the presence of a handler could have a negative impact on the interaction, in which case interacting with the dog alone would be more beneficial than interacting with the dog in the presence of the handler.

## 2. Materials and Methods

### 2.1. Participants

A total of 132 participants (85 females; mean age (*M*) = 21.6 years; range = 17–34 years; standard deviation (SD) = 3.4 years) were recruited via Heriot–Watt University Student Services voluntary sign-up system. The participants, all students, were assigned to one of three conditions on the day of the study. Forty-five participants took part in a standard Therapet session (TP), where participants interacted with both a handler and their dog; 41 participants were allocated to a control condition where participants interacted with a dog only (DO); with the remaining 46 participants taking part in a control condition in which the participants interacted with a handler only (HO). The participants in each of the three conditions were similar in terms of dog ownership and of pet ownership more generally. See Table 1 for a full breakdown of participant demographics, and pet ownership, by condition. 

### 2.2. Design

The study comprised a mixed design, with the TP, HO, or DO condition as the between-participants factor, and the pre- and post-completion of three questionnaires (measuring mood, anxiety, and well-being) as the within-participants factor. The participants completed each of the three questionnaires before being randomly allocated to one of three conditions for a 20 min session comprising either (1) a standard Therapet condition (TP) where participants interacted with both a dog and their handler; (2) a control condition (DO) in which the participants interacted with a dog only; or (3) a control condition (HO) in which the participants interacted with a handler only. Once the respective 20 min interaction period came to an end the participants completed the same three questionnaires that were presented pre-interaction.

### 2.3. Materials

The questionnaires used in the study included the Warwick–Edinburgh Mental Well-Being Scale (WEMWBS), the State-Trait Anxiety Inventory (STAI), and the UWIST Mood Adjective Check List (UMACL). In addition to completing the three questionnaires, the participants were asked to provide demographic information on age and gender, as well as information as to whether or not they are currently a dog owner or a pet owner more generally.

### 2.4. Animals

Seven Therapet dogs, and their handlers, took part in the study. The service, Therapet, was provided by Canine Concern Scotland Trust, which is a Scottish Charity No: SC014924, and runs Therapet sessions throughout Scotland. The dogs were Nina (a black Labrador), Mercy (Lhasa Apso), Pixie and Poppy (Cocker spaniels), Mack (Golden Retriever), Harvey (Collie-Spaniel), and Kiera (Border Collie). In any one session, 6 of the 7 dogs were present (according to availability), with approximately 6 participants “assigned” to a dog at any one time.

### 2.5. Procedure

#### 2.5.1. Pre-Session Measures

On arrival at the waiting area, the participants were taken to a quiet room adjacent to the large hall in which each session, irrespective of condition, would take place. The participants were subsequently briefed and asked to complete the three pre-test questionnaires (and provide demographic information). Upon completion of the questionnaires, the participants were informed as to which of the three conditions they had been allocated before being taken to the hall to undertake their session. In order to alleviate any disappointment associated with being allocated to the condition with no dog present (HO), those participants who were in the handler-only condition were assured that they would have an opportunity to interact with the dogs after their session was complete.

#### 2.5.2. Sessions

*Experimental Condition—Standard Therapet (TP)*. The experimental condition comprised a 20 min Therapet session identical to those that had been run on campus numerous times previously. The session was structured such that the participants could move freely between 6 stations, each of which comprised a (seated) handler and their dog, separated by enough space to allow the participants to sit in a semi-circle around the dog. The participants were told that they could ‘interact with any, or as many, dogs as they wished’ within the session; in return, the dog handlers were asked to interact freely with the participants and answer any questions that the participants posed to them (e.g., questions about the dog). 

*Control Condition—Dog Only (DO).* In the DO condition, the handlers were asked to limit their interaction with the participants to introducing themselves, and their dog, and explaining to the participants that they would not answer any questions during the session. In all other respects the set-up, procedure, and instructions provided to the participants were identical to those employed in the experimental condition.

*Control Condition—Handler Only (HO).* The participants in the HO condition interacted with the handlers only, the dogs were not present. The length and the structure of the session, and the handlers who took part, were identical to those employed in the two conditions in which dogs were present, with the exception that the participants were told that they could “interact with any, or as many, handlers as they wished”. The handlers were instructed to instigate similar conversational topics as if their dog was present. 

#### 2.5.3. Post-Session Measures

After the 20 min interaction was complete, the participants were taken back to the quiet room and asked to complete the questionnaires presented in the pre-session once more, before being fully debriefed. Those participants who took part in the handler-only condition were then allowed an opportunity to attend a standard Therapet session.

### 2.6. Ethics Approval

The study was approved by the Ethics Committee at Heriot Watt University Edinburgh (approval code: 2015-139), and was conducted in accordance with the Helsinki Declaration. All human participants signed an informed consent before taking part in the study. Approval from an animal ethics committee was not sought, as the data was collected during the regular, on-campus, Therapet activities. 

## 3. Results

### 3.1. Overview of Analysis

The key comparison in the analysis that follows is in the degree of shift witnessed in the pre- to post-test measures of mood, anxiety, and well-being in each condition. In order to make this comparison, we calculated a pre- to post-test shift score for each participant by subtracting the score obtained in each of the three post-test measures, from that obtained in the equivalent measure pre-test. The generation of a shift score for the participants on each of the three measures allowed us to directly compare the extent of the change across the three conditions using a MANOVA with condition as a between-participants factor. Preliminary analyses revealed: (1) that there was no significant difference in the participants’ well-being, anxiety, and mood pre-session scores in each of the three conditions (see Table 2, Table 3 and Table 4); and (2) that there was no influence of participant age, participant gender, dog ownership, or pet ownership on performance on all three measures, so these factors were not considered in the subsequent analysis. All analysis were conducted using SPSS 22 (IBM Corp, New York, NY, USA). In each analysis that follows effect sizes of 0.01 were deemed to be low, 0.06 intermediate, and 0.14 as high [24].

### 3.2. Influence of the Presence of a Dog

In order to determine whether the degree of change witnessed between the pre- and post-test measures differed significantly across conditions the change in the participants’ pre- and post-test scores for each of the three measures (mood, well-being, and anxiety) was subjected to a MANOVA with condition (TP, DO, or HO) as a between-participants factor. The analysis revealed that there was a significant difference between the three conditions across the three measures (*F*(6,246) = 10.48, *p* < 0.001; partial η^2^ = 0.26), with the between-participants effects, indicating that significant condition differences occurred with respect to mood (*F*(2,129) = 4.72, *p* = 0.011; partial η^2^ = 0.068), well-being (*F*(2,129) = 23.86, *p* < 0.001; partial η^2^ = 0.27), and anxiety (*F*(2,129) = 27.05, *p* < 0.001, partial η^2^ = 0.30; see Table 2, Table 3 and Table 4), and all three measures displaying high or intermediate effect sizes. With respect to mood, there were positive increases post-test in both of the conditions where a dog was present (TP *M* change = 2.62; DO *M* change = 4.15), a pattern of performance that differed from the handler-only condition where the shift in mood decreased post-test (HO *M* change = −0.26). Follow-up Bonferroni tests (not adjusted) revealed that the positive shift in mood witnessed in the dog-only condition was significantly greater than the change witnessed in the handler-only condition (*p* = 0.010). However, the difference between the handler-only condition, and the handler-plus-dog condition, was not significant, suggesting that interacting with a dog alone was particularly powerful in increasing mood, whereas including the interaction with the handler served only to reduce this positive influence. 

The positive influence of the presence of a dog was also evident with respect to well-being, where again positive shifts were evident post-test in both of the conditions in which dogs were present (TP *M* change = 2.36; DO *M* change = 1.78), which contrasted with performance in the handler-only condition, where a post-test decrease in well-being was witnessed (HO *M* change = −0.94). Follow-up Bonferroni tests revealed that the positive increase in well-being witnessed in both the DO and TP conditions was significantly greater than the change witnessed in the HO condition (*p* < 0.001 in each case), a finding that suggests that the presence of a dog (irrespective of handler presence) resulted in a positive shift in well-being. However, the difference between the two conditions in which dogs were present was not significant, suggesting that in comparison to the negative influence of the handler on the participants’ mood scores, the presence of the handler alongside the dog did not have a negative impact on well-being.

As with mood and well-being, the presence of a dog appeared to have a positive influence on the participant’s anxiety levels, with a large reduction in anxiety being witnessed post-test in both of the conditions in which dogs were present (TP *M* change = −13.73; DO *M* change = −12.98), with only a slight decrease in anxiety being witnessed in the handler-only condition (HO *M* change = −2.02). Follow-up Bonferroni tests revealed that the reduction in anxiety witnessed in both the DO and TP conditions was significantly greater than the reduction witnessed in the HO condition (*p* < 0.001 in each case), a finding that suggests that the presence of a dog (irrespective of whether the handler was present or not) resulted in a significant reduction in anxiety levels. The difference between the two conditions in which dogs were present was not significant, suggesting that the presence of the handler alongside the dog did not have a negative impact on well-being.

## 4. Discussion

The current study aimed to determine: (1) whether a one-off, dog-assisted, activity session improved the well-being, anxiety, and mood of university students, and (2), if so, which element of the activity was important in producing this effect, interacting with both the handler and the dog, interacting with the dog, or interacting with the handler. The results demonstrated that a short, 20 min session led to significantly greater pre–post interaction improvements in student well-being, and anxiety in both conditions where there was a dog present (irrespective of the presence of the handler), as compared to interacting with the handler alone. In contrast, interacting with the dog alone seemed to be most beneficial to mood, an overall pattern of performance which suggests that that there may not be one type of interaction that best fits all outcome measures. 

These results are broadly consistent with previous studies that have reported improvements in anxiety, mood, well-being, and perceived stress in college students after a therapy dog intervention [6,17,18,20,21]. Indeed, across our three measures, interacting with the dog appeared to be essential to the positive pre–post shift experienced by the students, with the presence of the handler having either a neutral (well-being and anxiety), or even a detrimental (mood) effect on outcome measures. Importantly, the more controlled nature of our study, in comparison to previous research, adds much needed data to the evidence base supporting the effectiveness of animal-assisted interventions [25,26,27]. This is, of course, an early attempt to dissect the usual animal-assisted activity and visitation programme structure, and one could argue that the separation of human handler, and therapy dog, in an intervention setting is somewhat artificial. However, our study demonstrates that this separation is crucial to detailing the most effective context in which to enhance different outcome measures, with moods appearing to differ from anxiety and well-being in the importance of dog/handler separation.

More broadly, the current findings indicate that animal-assisted interventions, activities, and visitation programmes can be successfully employed within higher education establishments to enhance student well-being and mental health. Such interventions have the potential to combat high stress levels, anxiety, and social isolation in the student population [14], and to increase the awareness and accessibility of counseling services within universities [15]. The positive benefits of allowing students to interact with dogs on campus is a relatively cost-effective way to enhance well-being and mental health, and has the clear potential to provide reference points to aid in making recommendations to services that work towards sustaining the mental health of student populations. This is of particular importance during periods of high pressure (e.g., exam time), as well as transitional periods where students are experiencing significant life changes (e.g., moving from home to university). Indeed, students who interact with dogs report feeling loved and accepted, factors that are key to optimal health during those high pressure and transitional periods. 

### Limitations and Further Recommendations

There are often general limitations associated with AAI and AAA studies, including inconsistent methodology, small sample sizes, and a lack of control groups [26,27]. We attempted to circumvent these limitations by including two control conditions alongside the standard Therapet condition. One such control required the participants to interact with the handler only, an allocation that was an important control, but had the potential to result in initial disappointment as the students would not be interacting with the dogs immediately. However, the participants in each of the three condition had similar baseline scores, and the participants were informed that they would interact with dogs immediately after their session, suggesting that the post-test measures were unlikely to have been affected by potential disappointment.

The self-selecting nature of the recruitment process also meant that specific individuals may have been more likely to engage with the study. In support of this claim, the final sample was skewed in favour of female participants, a pattern of responding that is consistent with a greater need in females to reduce anxiety [14]. This gender imbalance in no way diminishes the importance of the current findings but highlights the need to be acutely aware of the consequences of the recruitment process employed. Interestingly, the recruitment process appeared not to be skewed by dog or pet ownership (as may have been expected), with an approximately equal number of dog and other pet owners and non-owners taking part; a distribution of participants that nicely illustrates the powerful appeal of dog-assisted activities to a variety of individuals. 

Given the student sample, it is difficult to generalize the findings to other populations. However, students often experience challenges that are transferable to other groups including changes, transitions and high pressure, stressful, situations (e.g., exams, deadlines, social isolation and loneliness), suggesting that the current study lays important foundations for future research with a variety of non-clinical populations. Future studies should not only further dissect which elements of human–animal interaction impacts human health and well-being, they should include additional variables (e.g., neuro-physiological measures and direct vs. ambient interaction). In addition, future research should move beyond examining the “temporary relief” provided by a one-off session and incorporate longitudinal measures into their design.

## 5. Conclusions

This exploratory study advances previous research [28] by providing a controlled dissection of the benefits of each partner within the context of a dog visitation program, an approach that is in line with proposed routes to advancing research in human–animal interactions [6]. The study demonstrated that a short 20 min interaction with a therapy dog improved student well-being and mood and decreased anxiety levels (potentially removing impediments to learning). The study also demonstrated that it is the interaction with the therapy dog that affects these changes, with the handler providing a presence either neutral, or somewhat negative. The positive effects of animal-assisted interventions should be further explored in student populations, a group that is particularly susceptible to a variety of stressors that affect their resilience and mental health. Dog-assisted intervention appears to be a suitable, cheap, and effective alternative method to sustain student mental health and well-being.

## Figures and Tables

**Table 1:** Participant information and pet ownership in each of the three conditions.

Condition	*N*	Age (Years:Months)			Gender		Dog Owner		Pet Owner	
		*M*	SD	Range	Female	Male	Yes	No	Yes	No
TP (handler & dog interaction)	45	21.4	3.2	18–34	26	19	21	24	33	12
DO (dog only interaction)	41	21.7	3.6	18–34	31	10	23	18	25	16
HO (handler only interaction)	46	21.7	3.6	17–34	28	18	20	26	30	16

Note: *M* = mean age; SD = standard deviation.

**Table 2:** Mean score on the Mental Well-being Scale pre- and post-interaction in each of the three conditions (*N* = 132).

Condition	Pre Well-Being	Post Well-Being	Pre-Post Change
TP (handler & dog interaction)	46.33 ± 7.41 ^1^	48.69 ± 7.22	+2.36
DO (dog only interaction)	49.78 ± 7.91	51.56 ± 6.99	+1.78 **
HO (handler only interaction)	47.37 ± 7.57	46.43 ± 8.03	−0.94 **

Note: ^1^ Values represent mean ± standard deviation; ** *p* < 0.001.

**Table 3:** Mean score on the State-Trait Anxiety Inventory pre- and post-interaction in each of the three conditions (*N* = 132).

Condition	Pre Anxiety	Post Anxiety	Pre-Post Change
TP (handler & dog interaction)	43.00 ± 9.42 ^1^	29.27 ± 7.32	−13.73
DO (dog only interaction)	39.59 ± 10.71	26.61 ± 4.92	−12.98 **
HO (handler only interaction)	42.41 ± 10.81	40.39 ± 11.43	−2.02 **

Note: ^1^ Values represent means ± standard deviation; ** *p* < 0.001.

**Table 4:** Mean score on the Mood Adjective Check list pre- and post-interaction in each of the three conditions (*N* = 132).

Condition	Pre Mood	Post Mood	Pre-Post Change
TP (handler & dog interaction)	61.89 ± 5.87 ^1^	64.51 ± 3.49	+2.62
DO (dog only interaction)	62.24 ± 7.22	66.39 ± 3.71	+4.15 *
HO (handler only interaction)	63.50 ± 5.81	63.24 ± 6.51	−0.026

Note: ^1^ Values represent means ± standard deviation; * *p* < 0.05.
